# Impact of radiological honeycombing in rheumatoid arthritis-associated interstitial lung disease

**DOI:** 10.1186/s12890-020-1061-x

**Published:** 2020-01-30

**Authors:** Hideaki Yamakawa, Shintaro Sato, Tomotaka Nishizawa, Rie Kawabe, Tomohiro Oba, Akari Kato, Masanobu Horikoshi, Keiichi Akasaka, Masako Amano, Hiroki Sasaki, Kazuyoshi Kuwano, Hidekazu Matsushima

**Affiliations:** 1 Department of Respiratory Medicine, Saitama Red Cross Hospital, 1-5 Shintoshin, Chuo-ku, Saitama, 330-8553 Japan; 2 Department of Internal Medicine, Division of Respiratory Medicine, Jikei University School of Medicine, Tokyo, Japan; 3 Department of Rheumatology, Saitama Red Cross Hospital, Saitama, Japan; 4 Department of Radiology, Saitama Red Cross Hospital, Saitama, Japan

**Keywords:** Honeycombing, Interstitial lung disease, Prognosis, Rheumatoid arthritis, Time course

## Abstract

**Background:**

Interstitial lung disease (ILD) is the most common and important pulmonary manifestation of rheumatoid arthritis (RA). A radiological honeycomb pattern has been described in diverse forms of ILD that can impact survival. However, the clinical course and sequential radiological changes in the formation of the honeycomb pattern in patients with RA-ILD is not fully understood.

**Methods:**

We evaluated the sequential changes in computed tomography findings in 40 patients with chronic forms of RA-ILD without the honeycomb pattern at initial diagnosis. We classified the patients into the Non-honeycomb group and Honeycomb group, and then analyzed the characteristics and prognosis of the two groups. The term “honeycomb formation” indicated a positive finding of honeycombing on any available follow-up CT.

**Results:**

Our RA-ILD cohort included patients with probable usual interstitial pneumonia (UIP) (35%), nonspecific interstitial pneumonia (NSIP) (20%), and mixed NSIP/UIP (45%). Among all RA-ILD patients, 16 (40%) showed honeycomb formation on follow-up CT (median time between initial and last follow-up CT was 4.7 years). Patient characteristics and prognosis were not significantly different between the Non-honeycomb and Honeycomb groups. However, Kaplan-Meier survival curve for the time from the date of honeycomb formation to death showed a poor median survival time of 3.2 years.

**Conclusions:**

A certain number of patients with RA-ILD developed a honeycomb pattern during long-term follow-up, regardless of whether they had UIP or NSIP. Prognosis in the patients with characteristics of both progressive ILD and honeycomb formation could be poor. Although radiological findings over the disease course and clinical disease behavior in RA-ILD are heterogenous, clinicians should be alert to the possibility of progressive disease and poor prognosis in patients with RA-ILD who form a honeycomb pattern during follow-up observation.

## Introduction

Interstitial lung disease (ILD) is a progressive fibrotic disease of the lung parenchyma. Occurring in association with several connective tissue diseases, it is the most common and important pulmonary manifestation of rheumatoid arthritis (RA) [1]. The poor prognostic factors of RA-ILD include male sex, older age, a wide range of fibrotic changes on computed tomography (CT), usual interstitial pneumonia (UIP) pattern, and acute exacerbation [2–5]. In particular, radiological analysis by high-resolution CT (HRCT) is often used in clinical practice, and for clinicians determining the management of RA-ILD, it is important to know whether each patient has UIP [5–7]. However, an unclassifiable pattern on HRCT is present to some extent in the RA-ILD population because RA-ILD exhibits a diversity of patterns [6, 8]. Recent reports including our study showed that the major HRCT pattern in RA-ILD was mixed nonspecific interstitial pneumonia (NSIP) and UIP [9, 10]. However, radiological honeycomb pattern is a poorer prognostic factor than a UIP pattern on HRCT [10]. Adegunsoye et al. recently noted that honeycomb represents a progressive fibrotic ILD phenotype of underlying disease such as RA and chronic hypersensitive pneumonia [11]. Therefore, we highlight radiological honeycomb as the most important radiological finding. Further, the radiological course of honeycomb formation over long-term follow-up is not well known. Thus, the aims of the present study were to assess the sequential radiological changes (i.e., whether honeycombing formed) in patients with RA-ILD and to evaluate whether this impacts their survival.

## Materials and methods

### Study sample

Patients with chronic RA-ILD diagnosed from January 2012 to December 2017 and followed at our institution were reviewed for initial and subsequent CT studies for at least one year after diagnosis of RA-ILD based on CT findings. Only patients with RA-ILD and without honeycomb at the initial diagnosis of RA-ILD were analyzed. A diagnosis of RA was made in these patients according to the American College of Rheumatology/European League Against Rheumatism Criteria by rheumatology specialists [12]. We then collected data from each patient’s medical records that included characteristics, laboratory data, pulmonary function results, and chest CT findings at the time of ILD diagnosis. This patient cohort was already the subject of a previous study focusing on interstitial pneumonia with RA [10]. This study was approved by the institutional review board of Saitama Red Cross Hospital (approval no. 18-AE).

### Data collection

Baseline clinical measurements were obtained within 3 months of the initial diagnosis of ILD. Radiological images were independently reviewed by one experienced radiologist (H.S.) and one experienced pulmonologist (H.M.) blinded to the patients’ clinical information. HRCT patterns were classified as probable UIP, indeterminate (mixed NSIP/UIP), or NSIP according to our recent modified guidelines for idiopathic pulmonary fibrosis (IPF) [10, 13]. For combined pulmonary fibrosis with emphysema, positive findings of emphysema were visually defined as the presence of a low attenuation indicating the lack of a distinct alveolar wall threshold over 10% [14]. Honeycomb is defined as clustered cystic air spaces with typically comparable diameters of 3–10 mm in subpleural and lower lobes with well-defined walls [13]. The term “honeycomb formation” indicated a positive finding of honeycombing on any available follow-up CT. Follow-up CT findings were evaluated by the same radiologist and pulmonologist. CT scans were assessed for Kappa interrater agreement, after which discordant studies were reviewed collaboratively for consensus agreement. Acute exacerbation of RA-ILD was defined based on a previous study [3, 10].

### Statistical methods

The observation follow-up period was calculated from the date of initial ILD diagnosis until the patient’s last visit or time of death. The follow-up period of CT scans was calculated from the date of initial ILD diagnosis until last date of an available CT. Categorical baseline characteristics are summarized by frequency and percentage, and continuous characteristic are reported as mean ± SD. To detect differences between groups, the unpaired *t*-test, Fisher’s exact test, or Mann-Whitney U test was used as appropriate. The Kaplan-Meier method and log-rank test were used to display and compare survival curves for the cohort stratified for each group. In addition, a Kaplan-Meier survival curve was created for the time from the date of honeycomb formation to death in the Honeycomb group. We considered *P* < 0.05 to indicate statistical significance. All data were analyzed with SPSS version 22.0 (IBM Japan, Tokyo, Japan).

## Results

### Patient characteristics

**Fig. 1:**
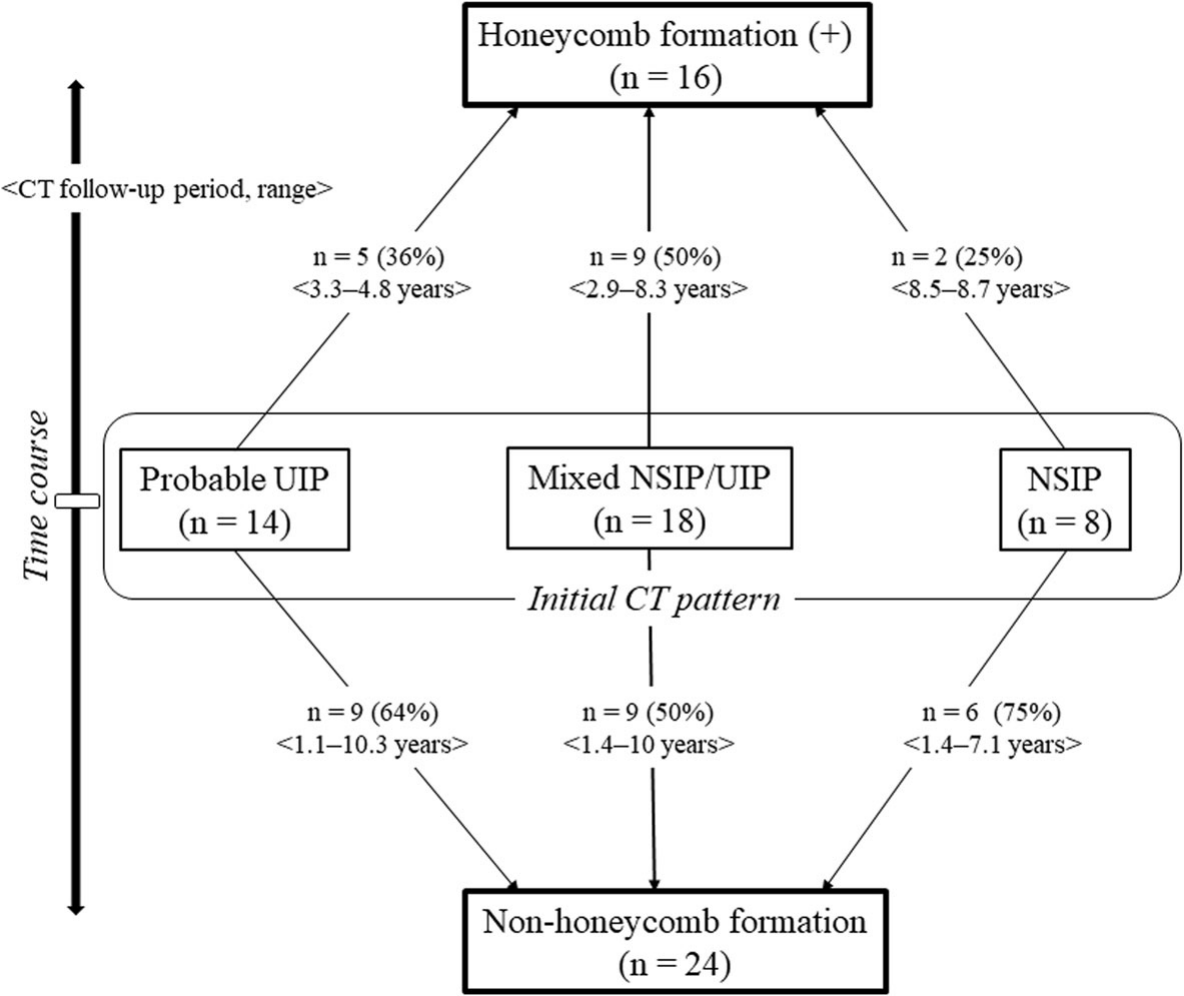
Radiological time course of the patients with RA-ILD. Abbreviations: ILD: interstitial lung disease; NSIP: nonspecific interstitial pneumonia; RA: rheumatoid arthritis; UIP: usual interstitial pneumonia

Among 78 patients with chronic RA-ILD, 26 patients with honeycomb lung at the initial diagnosis were excluded. In addition, 12 patients having at least two CT scans more than 1 year apart were also excluded. Thus, the remaining 40 patients were included in our study, The initial CT patterns were probable UIP in 14 patients, mixed NSIP/UIP in 18 patients, and NSIP in 8 patients (Fig. 1).

**Fig. 2:**
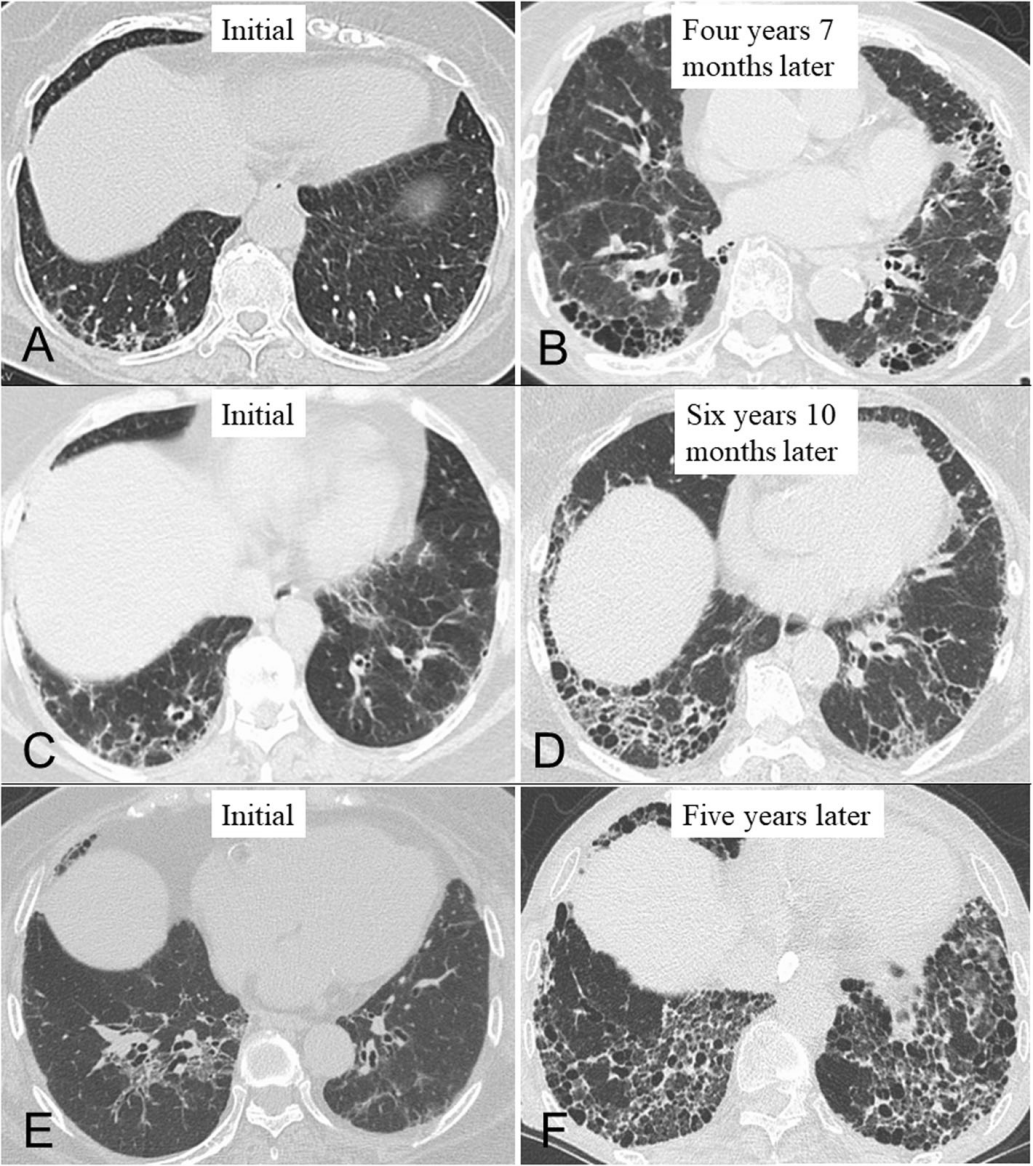
Computed tomographic images illustrating each pattern in the evolution of honeycombing in rheumatoid arthritis with interstitial lung disease during the follow-up period. (**a**/**b**) Radiological time-dependent changes in a 79-year-old woman with probable usual interstitial pneumonia (UIP) pattern. (**c**/**d**) Radiological time-dependent changes in a 63-year-old woman with mixed nonspecific interstitial pneumonia (NSIP)/UIP pattern. (**e**/**f**) Radiological time-dependent changes in a 78-year-old woman with NSIP pattern

Among all patients, the median follow-up period between the initial and last CT was 4.7 years, and 16 patients (40%) evolved a honeycomb pattern (Fig. 1). Based on the evolution of honeycombing on any available follow-up CT, interobserver agreement regarding honeycomb formation was moderate (Kappa value = 0.57, 95% confidence interval 0.296–0.835). Honeycombing developed in 5 patients (36%) with an initial diagnosis of probable UIP (Fig. 2a/b), 9 patients (50%) with mixed NSIP/UIP (Fig. 2c/d), and 2 patients (25%) with NSIP (Fig. 2e/f).

**Fig. 3:**
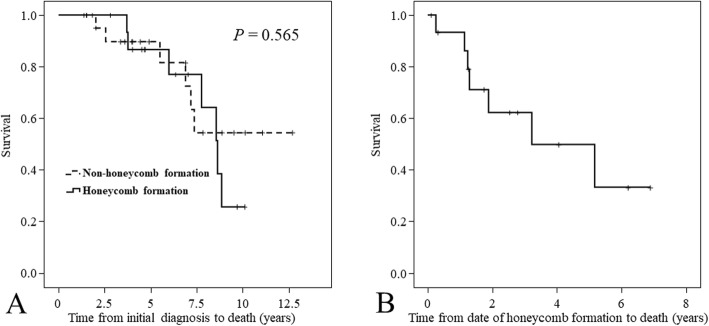
Kaplan-Meier survival curves. (**a**) Kaplan-Meier survival curve from the initial diagnosis to death in patients with non-honeycomb formation (*N* = 24) versus those with honeycomb formation (*N* = 16). Patient characteristics and prognosis were not significantly different between the two groups (log rank, *P* = 0.565). (**b**) Kaplan-Meier survival curve from the time of honeycomb formation to death in the group with honeycomb formation showed a median survival time of 3.2 years and 5-year survival rate of 49.8%

The median follow-up period between the initial and last CT scan was 5.2 years in the Honeycomb group versus 4.0 years in the Non-honeycomb group (*P* = 0.194) (Table 1). There were no significant differences between the two groups in terms of initial patient characteristics including sex, age, past smoking history, chest radiological findings, Krebs von den Lungen-6 and surfactant protein-D levels, pulmonary function findings, and medications (Table 1). The Kaplan-Meier survival curves of the two groups are shown in Fig. 3a. Cumulative 5-year survival rates were 89.7% in the Non-honeycomb group and 86.7% in the Honeycomb group, and then survival periods were not significantly different (log rank *P* = 0.565). The Kaplan-Meier survival curve for the time from the date of honeycomb formation to death in the Honeycomb group showed a median survival time of 3.2 years and 5-year survival rate of 49.8% (Fig. 3b).

**Table 1:** Characteristics of and medications for RA-ILD

	Non-honeycomb group	Honeycomb group	*P* value
No. of patients	24	16	
Age, years, mean ± SD	65.3 ± 11.6	67.9 ± 7.4	0.443
Male, N (%)	8 (33%)	8 (50%)	0.339
Current or ex-smoker, N (%)	11 (46%)	9 (56%)	0.748
Initial CT pattern, N (%)			0.491
Probable UIP	9 (38%)	5 (31%)	
Indeterminate; mixed NSIP/UIP	9 (38%)	9 (56%)	
NSIP	6 (25%)	2 (13%)	
CPFE, N (%)	7 (29%)	2 (13%)	0.272
Acute exacerbation of ILD during follow-up, N (%)	4 (17%)	3 (19%)	> 0.999
KL-6, U/mL, mean ± SD	804.7 ± 525.9	1150.1 ± 791.4	0.114
SP-D, ng/mL, mean ± SD	144.3 ± 121.0	172.2 ± 136.4	0.610
%FVC, mean ± SD	84.5 ± 20.2	82.8 ± 14.2	0.812
FEV_1_/FVC ratio, % mean ± SD	81.5 ± 7.8	86.6 ± 6.8	0.122
%DL_CO_, mean ± SD	67.5 ± 14.8	62.2 ± 12.5	0.420
CPI, mean ± SD	32.4 ± 12.1	41.0 ± 11.3	0.138
Median follow-up period of CT scans, years (range)	4.0 (1.1–10.3)	5.2 (2.9–8.7)	0.194
Median observation follow-up, years (range)	4.7 (1.4–12.7)	6.2 (2.8–10.1)	0.269
Deaths (during follow-up), N	6 (25%)	7 (44%)	0.305
Cause of death, N			0.643
Chronic respiratory failure	1	2	
Acute exacerbation	3	1	
Malignancy	1	1	
Others	1	3	
Medications used during follow-up, N			
Corticosteroid	9	11	0.105
Methotrexate	9	3	0.297
Iguratimod	3	0	0.262
Calcineurin inhibitor	9	7	0.750
Biologics	5	2	0.681
Pirfenidone or nintedanib	0	1	0.400

*RA* rheumatoid arthritis; *ILD* interstitial lung disease; *SD* standard deviation; *CTD* connective tissue disease; *CT* computed tomography; *UIP* usual interstitial pneumonia; *NSIP* nonspecific interstitial pneumonia; *CPFE* combined pulmonary fibrosis with emphysema; *KL-6* Krebs von den Lungen-6; *SP-D* surfactant protein-D; *FVC* forced vital capacity; *FEV*_*1*_ forced expiratory volume in 1 s; *DL*_*CO*_ diffusing capacity of the lung for carbon monoxide; *CPI* composite physiological index

## Discussion

Radiological honeycombing has been described in diverse forms of ILD, but its prevalence and association with mortality across the spectrum of ILD remain unclear [11]. The present study aimed to assess the time course over which radiological honeycombing could evolve and whether its formation would influence survival in patients with RA-ILD.

First, in terms of radiological changes occurring during the follow-up period (median duration: 4.7 years), 40% of the RA-ILD patients formed honeycombing. Yamauchi et al. reported that in IPF, 53.3% of the patients developed honeycombing over a mean follow-up period of 5.9 years [15]. Giacomi et al. also reported the development of honeycombing in 32% of their patients over a median follow-up period of 4.8 years [16]. The present study is, to our knowledge, the first to focus on the development of honeycombing during follow-up for RA-ILD. In our cohort, 36% of patients with probable UIP and 50% of patients with mixed NSIP/UIP developed honeycombing, and to some extent, patients with RA-ILD developed honeycombing during long-term follow-up as a component of IPF. Importantly, over the long term, honeycomb also arose in a quarter of the RA-ILD patients with NSIP. In 28% of idiopathic ILD patients with initial findings suggestive of NSIP, follow-up CT scans were interpreted as more suggestive of IPF [17]. Taken together, we observed that a certain number of chronic ILD patients developed honeycombing over the long term, regardless of their underlying disease (i.e., RA or idiopathic) and CT pattern (i.e., probable UIP, mixed NSIP/UIP, or NSIP).

Second, we found no significant difference in the prognoses of the RA-ILD patients who did or did not eventually develop honeycombing. In IPF, it is controversial whether having honeycomb is a poor prognostic factor [11, 15]. However, recent reports indicated that the development of honeycombing in RA-ILD was a poorer prognostic factor than CT pattern (e.g., UIP, NSIP) [9, 11]. Similarly, our recent study also showed having honeycomb to be a poor prognostic factor in RA-ILD [10]. Therefore, we speculated that the small sample size in our study probably induced this result. In fact, the Kaplan-Meier survival curve for the time from the date of honeycomb formation to death in the Honeycomb group showed a poor median survival time of 3.2 years. In comparison with this survival time, surprisingly, our previous study showed a median survival time of 6.4 years in the RA-ILD patients with honeycomb. Therefore, it appears that survival is poorer in RA-ILD patients who develop a honeycomb pattern over the disease course than in those with honeycomb found at the initial diagnosis [10]. Thus, the possibility that patients with the characteristics of both progressive ILD and honeycomb development must be considered to have a poor prognosis. However, both our previous and present studies present the possibility that the disease course in some RA-ILD patients with honeycomb at the initial diagnosis might stabilize as burnt out. A minority of patients, even those with a UIP pattern, experience significant improvement or stabilization in pulmonary function over the disease course [18]. Taken together, the disease behavior of RA-ILD accompanied by honeycombing is heterogeneous, and the most important thing is to evaluate the disease, including radiological changes, over the entire clinical course rather than only at specific points.

There are limitations in the current study. First, it is a retrospective study with a relatively small number of patients. Second, selection bias may be present because this study was performed in a single center, and some of the patients were excluded from the long-term analysis due to insufficient follow-up data. Third, we could not determine whether the clinical diagnosis of RA-ILD impacted treatment decisions and, as such, the natural disease course.

## Conclusions

The present study confirmed that radiological findings over the disease course and clinical disease behavior in RA-ILD are heterogenous. A certain number of RA-ILD patients developed a honeycomb pattern over long-term follow-up, regardless of whether they had UIP or NSIP. A progressive disease course and radiological honeycombing could be useful predictors of poor prognosis in patients with RA-ILD. This may help in assessing appropriate strategies to treat the RA itself and the combination of RA and ILD.

## Data Availability

The datasets used and/or analyzed during the current study are available from the corresponding author on reasonable request.

## Abbreviations

CT: Computed tomography
HRCT: High-resolution computed tomography
ILD: Interstitial lung disease
IPF: Idiopathic pulmonary fibrosis
NSIP: Nonspecific interstitial pneumonia
RA: Rheumatoid arthritis
UIP: Usual interstitial pneumonia
